# Interleukin-6 regulates iron-related proteins through c-Jun N-terminal kinase activation in BV2 microglial cell lines

**DOI:** 10.1371/journal.pone.0180464

**Published:** 2017-07-03

**Authors:** Shida Zhou, Xinxing Du, Junxia Xie, Jun Wang

**Affiliations:** 1 Department of Physiology, Shandong Provincial Key Laboratory of Pathogenesis and Prevention of Neurological Disorders and State Key Disciplines: Physiology, Medical College of Qingdao University, Qingdao, China; 2 Class 7, Grade 2014, Medical College of Qingdao University, Qingdao, China; Universidade de Sao Paulo, BRAZIL

## Abstract

Parkinson’s disease (PD) is a common neurodegenerative disorder characterized by the loss of dopaminergic (DA) neurons in the substantia nigra (SN) and subsequent DA depletion in the striatum. Microglia activation and nigral iron accumulation play important roles in the pathogenesis of PD. Activated microglia show increased iron deposits. However, the relationship between microglia activation and iron accumulation remains unclear. In the present study, we aimed to determine how iron levels affect interleukin-6 (IL-6) synthesis, and the effect of IL-6 on cellular iron metabolism in BV2 microglial cells.IL-6 mRNA was up-regulated after FAC treatment for 12 h in BV2 cells. Iron regulatory protein 1 (IRP1) and divalent metal transporter 1 (DMT1) were up-regulated and iron exporter ferroportin 1 (FPN1) was down-regulated in BV2 cells after 24 h of IL-6 treatment. Phosphorylated JNK increased significantly compared to the control after BV2 cells were treated with IL-6 for 1 h. Pretreatment with SP600125 attenuated the up-regulation of IRP1 and DMT1 and down-regulation of FPN1 (compared to IL-6-treated group). These results suggest that iron load could increase IL-6 mRNA expression in BV2 cells. Further, IL-6 likely up-regulates IRP1 and DMT1 expression and down-regulates FPN1 expression in BV2 microglial cells through JNK signaling pathways.

## Introduction

Parkinson’s disease (PD) is a common neurodegenerative disorder characterized by the loss of dopaminergic neurons in the substantia nigra (SN) and subsequent dopamine (DA) depletion in the striatum. Microglia activation has been observed in patients with PD. Reactive microglia become neurotoxic through the secretion of inflammatory cytokines such as tumor necrosis factor alpha (TNF-α), interleukin 1 beta (IL-1β), and interleukin 6 (IL-6)[1–3]. IL-1β, IL-6, and TNF-α levels increase in the brain of patients with PD [4].

Extensive studies have demonstrated that iron plays a key role in the pathogenesis of PD. Increased expression of the iron import protein divalent metal transporter1 (DMT1) and decreased expression of the iron exporter ferroportin 1 (FPN1), caused by increased expression of iron regulatory protein 1 (IRP1), results in increased iron levels [5]. IRP1 is a central regulator of iron homeostasis, and is likely a target of extracellular agents that regulate changes in cellular iron metabolism. FPN1 mRNAs contain an iron responsive element (IRE) in the 5′-untranslated region (UTR), where binding of IRP could interfere with the initiation of translation, leading to decreased FPN1 expression [5, 6]. According to the IRE/IRP theory [5], IRP1 binds to the IRE in the 3′-UTR of DMT1+IRE mRNA, and can thereby increase DMT1 stability, leading to increased DMT1 expression.

Microglia activation and nigral iron accumulation were both found in the SN of 1-methyl-4-phenyl-1,2,3,6-tetrahydropyridine (MPTP), 6-hydroxydopamine (6-OHDA), and rotenone-induced PD animal and cell models. The activated microglia showed increases in iron deposits. However, the relationship between microglia activation and iron accumulation remains unclear. Our previous study [7] showed that inflammatory cytokines such as TNF-α and IL-1β induced iron accumulation in neurons by up-regulating DMT1 and down-regulating FPN1 in vitro. Therefore, we aimed to determine how iron levels affect inflammatory cytokine synthesis and the effect of inflammatory cytokines on iron metabolism in microglial cells.

## Materials and methods

### Materials

Ferric ammonium citrate (FAC) and SP600125 were obtained from Sigma Chemical Co. (St Louis, MO, USA). Dulbecco's modified Eagle's medium (DMEM) and heat-inactivated fetal bovine serum were obtained from Gibco (NY, USA). Trizol Reagent was purchased from Invitrogen (CA, US). The reverse-transcription system was purchased from Thermo (CA, US), and the SYBR Green system from Qiagen (Hilden, Germany). IRP1, DMT1, and secondary antibody conjugated to horseradish peroxidase antibodies were obtained from Santa Cruz (CA, US). FPN1 antibody was obtained from Sigma (Darmstadt, Germany). JNK and Phospho-JNK were purchased from Cell Signaling Technology (MA, US). ß-Actin antibody was obtained from Bioss (Beijing, China). All other chemicals and regents were of the highest grade available and were obtained from local commercial sources.

### Cell culture

BV2 microglia cells were purchased from the China Infrastructure of Cell Line Resources (Beijing, China). For experiments, cells were cultured in 12-well cell culture plates at a density of 1 × 10^5^ cells/cm^2^ in Dulbecco’s Modified Eagle Medium supplemented with 10% heat-inactivated fetal bovine serum, penicillin G (100 units/mL), and streptomycin (100 mg/mL), and were incubated at 37°C in a humidified atmosphere containing 5% CO_2_.

### Drug treatment

BV2 microglia cells cultured in DMEM were treated for 24 h with 100 μmol/L FAC to study the expression of IL-6 mRNA, as described previously [8]. To detect phosphorylated JNK/ total JNK ratio changes, BV2 microglia cells were treated with 30 ng/ml IL-6 for 1 h [9].

To determine the effect of IL-6 on iron metabolism-related proteins in BV2 microglial cell lines, BV2 microglia cells were divided into 3 groups: (1) normal control; (2) IL-6, wherein cells were stimulated with 30 ng/ml IL-6 for 24 h; and (3) SP600125+IL-6 30 ng/ml, wherein cells were preincubated with 20 μmol/L SP600125, a JNK inhibitor, for 1 h and then treated with 30 ng/ml IL-6 for 24 h.

### Total RNA extraction and real-time PCR

Total RNA was isolated from BV2 microglia cells by using trizol reagent, according to the manufacturer’s instructions. Total RNA (2 μg) was reverse-transcribed in a 20-μL reaction with oligo-dT primers by using a reverse-transcription system. cDNA expression was assayed by real-time PCR performed using the SYBR Green system. Specific primers were designed as follows: IL-6 forward 5′-CCACTTCACAAGTCGGAGGCTTA-3′, reverse 5′-GCAAGTGCATCATCGTTGTTCATAC-3′; GAPDH forward 5′-TGTGTCCGTCGTGGATCTGA-3′, reverse 5′-TTGCTGTTGAAGTCGCAGGAG-3′. Amplification and detection were performed under the following conditions: an initial hold at 95°C for 5 min followed by 40 cycles at 95°C for 10 s and 60°C for 30 s.

### Western blots

After three washes with cold PBS, cells were lysed in lysis buffer with protease inhibitors (1 mmol/L phenylmethylsulfonyl fluoride) for 30 min on ice and centrifuged for 15 min at 12000 g at 4°C. Total proteins were separated by 10% SDS-polyacrylamide gel electrophoresis and transferred to polyvinylidene fluoride (PVDF) membranes. After 2 h of blocking with 10% non-fat milk at room temperature, the membranes were incubated at 4°C overnight with primary antibodies against IRP1 (1:1000), DMT1 (1:800), FPN1 (1:1000), JNK (1:1000), Phospho-JNK (1:1000), and β-Actin (1:8000). Secondary antibody conjugated to horseradish peroxidase (1:10000) was used at 1:10000. Cross-reactivity was visualized using ECL western blotting detection reagents and analyzed through scanning densitometry

### Statistical analysis

Statistical analyses were performed using SPSS software for Windows (version 19.0). Data are presented as the means ± SEM. Differences between means in two groups were compared using the unpaired-samples *t*-test. One-way analysis of variance (ANOVA) was used to test for multiple comparisons. P < 0.05 was considered statistically significant.

## Results

### IL-6 mRNA expression was enhanced in FAC-treated BV2 cells

**Fig 1:**
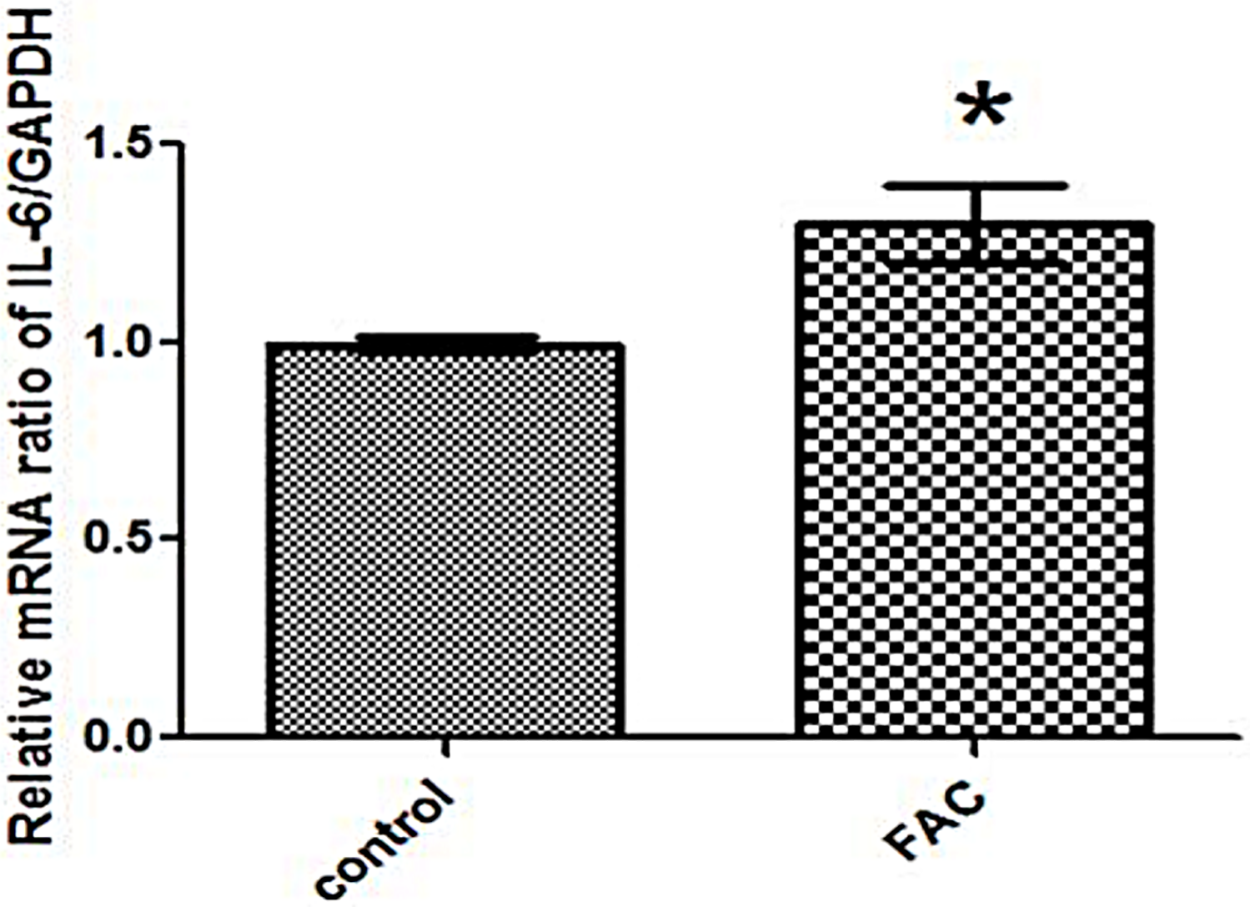
IL-6 mRNA was up-regulated after FAC treatment in BV2 cells. BV2 microglial cells were treated with FAC for 24 h. Real-time PCR was used to detect IL-6 mRNA levels. IL-6 mRNA was up-regulated after FAC treatment compared with the untreated control. Each bar represents the mean ± S.E.M of 6 independent experiments (*P < 0.05 compared with the control; n = 6).

We first ascertained whether iron levels affect IL-6 synthesis in BV2 microglia. BV2 microglial cells were treated with 100 μmol/L FAC for 24 h. As shown in Fig 1, IL-6 mRNA significantly increased in FAC-treated microglia compared to the control (Fig 1), suggesting that IL-6 mRNA levels increased in microglia under conditions of iron load.

### IL-6 affected the expression of iron-metabolism-related proteins in BV2 cells

**Fig 2:**
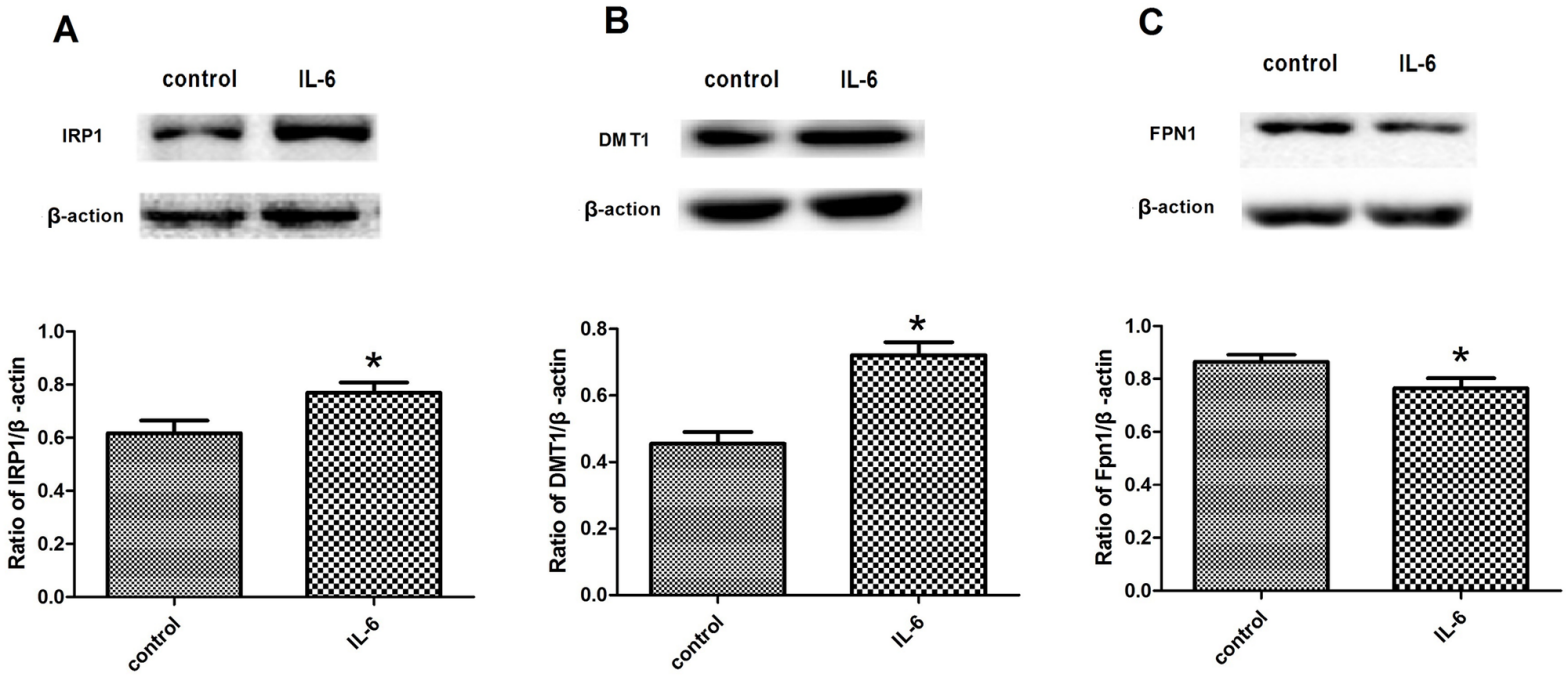
Expression of IRP1, DMT1, and FPN1 in BV2 cells treated with IL-6. A: Western blots to detect IRP1 expression. IRP1 expression was significantly increased in BV2 cells treated with IL-6. B: Western blots to detect DMT1 expression. DMT1 expression was significantly increased in BV2 cells treated with IL-6. C: Western blots to detect FPN1 expression. FPN1 expression was significantly decreased in BV2 cells treated with IL-6. All data are presented as the ratio of iron-related-proteins to β-actin. Each bar represents the mean ± S.E.M of 5 independent experiments (*P < 0.05 compared with control; n = 5).

To determine whether the levels of iron transporters were responsive to IL-6 treatment in microglia, we investigated the expressions of IRP1, DMT1, and FPN1 in BV2 cells treated for 24 h with 30 ng/ml IL-6. IRP1 and DMT1 expression increased, and FPN1 expression decreased in the IL-6-treated BV2 cells (Fig 2). Thus, IL-6 up-regulated DMT1 expression and down-regulated FPN1 expression by up-regulating IRP1, and could thereby cause an increase in iron level in BV2 cells.

### IL-6 induced up-regulation of the phosphorylated JNK/ total JNK ratio in BV2 cells

**Fig 3:**
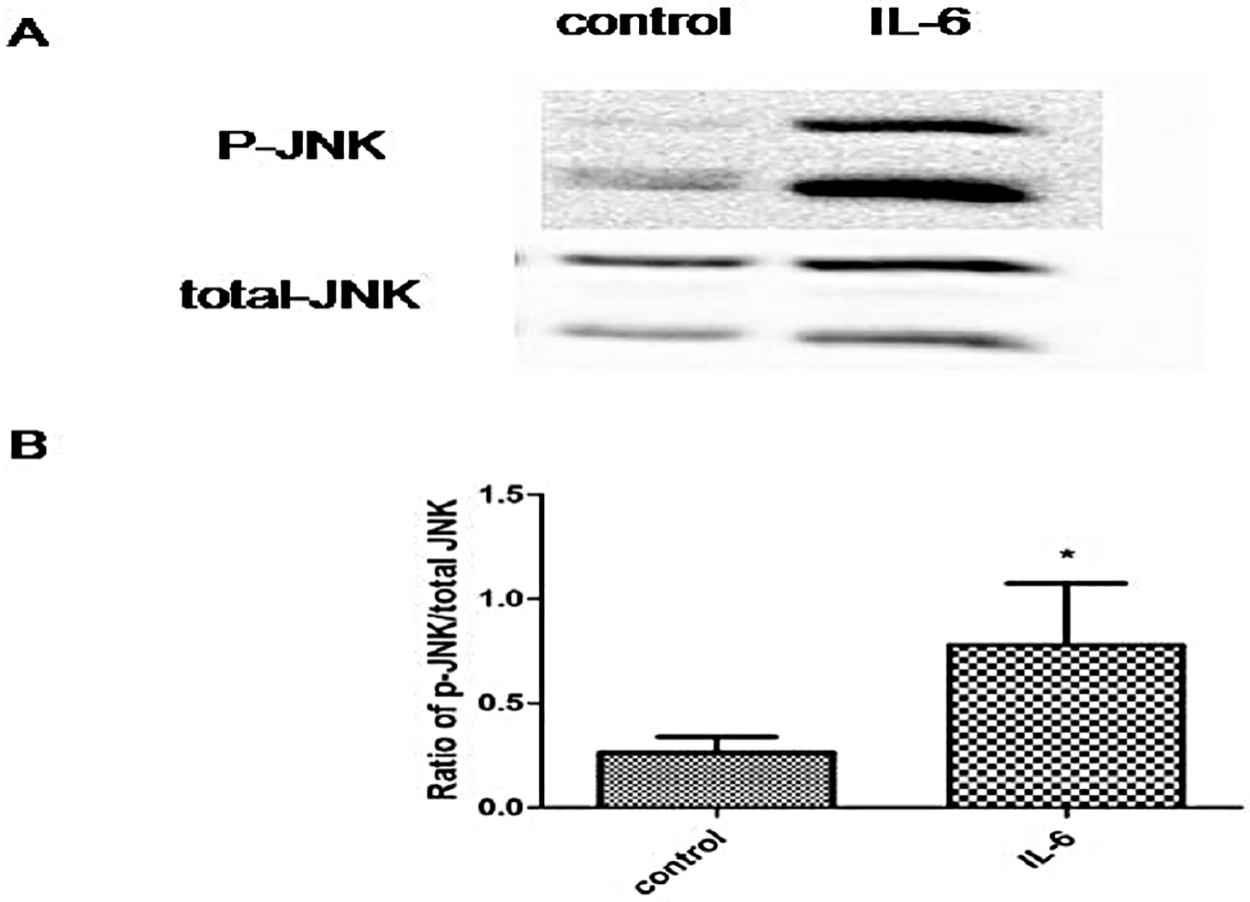
Phosphorylated JNK/total JNK ratios were increased in IL-6-treated BV2 cells. A: Western blots to detect phosphorylated JNK and total JNK expression. Ratio of phosphorylated JNK/total JNK increased after IL-6 treatment for 1 h, compared with the control group. B: Statistical analysis. Data are presented as the ratio of P-JNK to total JNK. Each bar represents the mean ± S.E.M of 3 independent experiments (*P < 0.05 compared with the control; n = 3).

Extensive evidence suggests that JNK forms a complex network to regulate iron metabolism. JNK is therefore a possible link between IL-6 and iron metabolism. Treatment of BV2 cells with 30 ng/ml IL-6 for 1 h resulted in a significant increase in the phosphorylated JNK/ total JNK ratio compared with the control group, indicating that IL-6 activated JNK in BV2 cells (Fig 3).

### IL-6 regulates the expression of iron-metabolism-related proteins by activating JNK pathways

**Fig 4:**
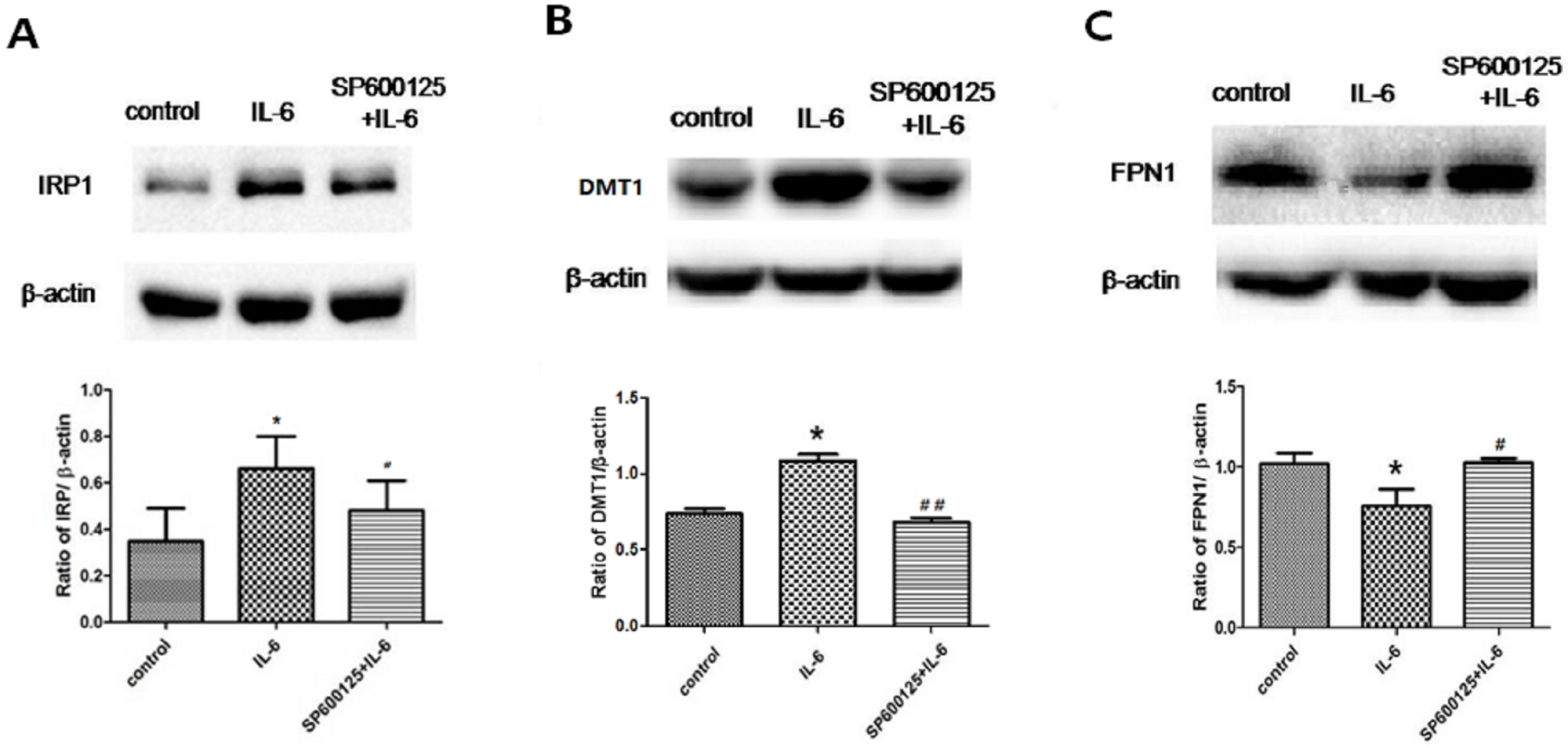
Expression of IRP1, DMT1, and FPN1 in IL-6-treated BV2 cells was inhibited by SP600125 pretreatment. A: Western blots to detect IRP1 expression. IRP1 expression was significantly increased in BV2 cells treated with IL-6. Pretreatment with SP600125 attenuated the up-regulation of IRP1. B: Western blots to detect DMT1 expression. DMT1 expression was significantly increased in BV2 cells treated with IL-6. Pretreatment with SP600125 attenuated the up-regulation of DMT1. C: Western blots to detect FPN1 expression. FPN1 expression significantly decreased in BV2 cells treated with IL-6. Pretreatment with SP600125 attenuated the down-regulation of FPN1. β-actin was used as a loading control. Data are presented as the ratio of protein to β-actin. Each bar represents the mean ± S.E.M of 4 independent experiments (*P < 0.05 compared with control, ^#^P < 0.05 compared with IL-6 group; n = 4).

To determine whether IL-6 affects iron metabolism via the JNK signaling pathway, we pre-treated the BV2 cells with SP600125 to inhibit JNK phosphorylation. As shown in Fig 4A, the expression of IRP1 was significantly increased in BV2 cells treated with IL-6, and pretreatment with SP600125 attenuated the up-regulation of IRP1. In addition, the up-regulation of DMT1 and down-regulation of FPN1 were inhibited by pretreatment with SP600125 (Fig 4B and 4C). Thus, IL-6 regulated some iron-metabolism-related proteins through JNK signaling pathways.

## Discussion

**Fig 5:**
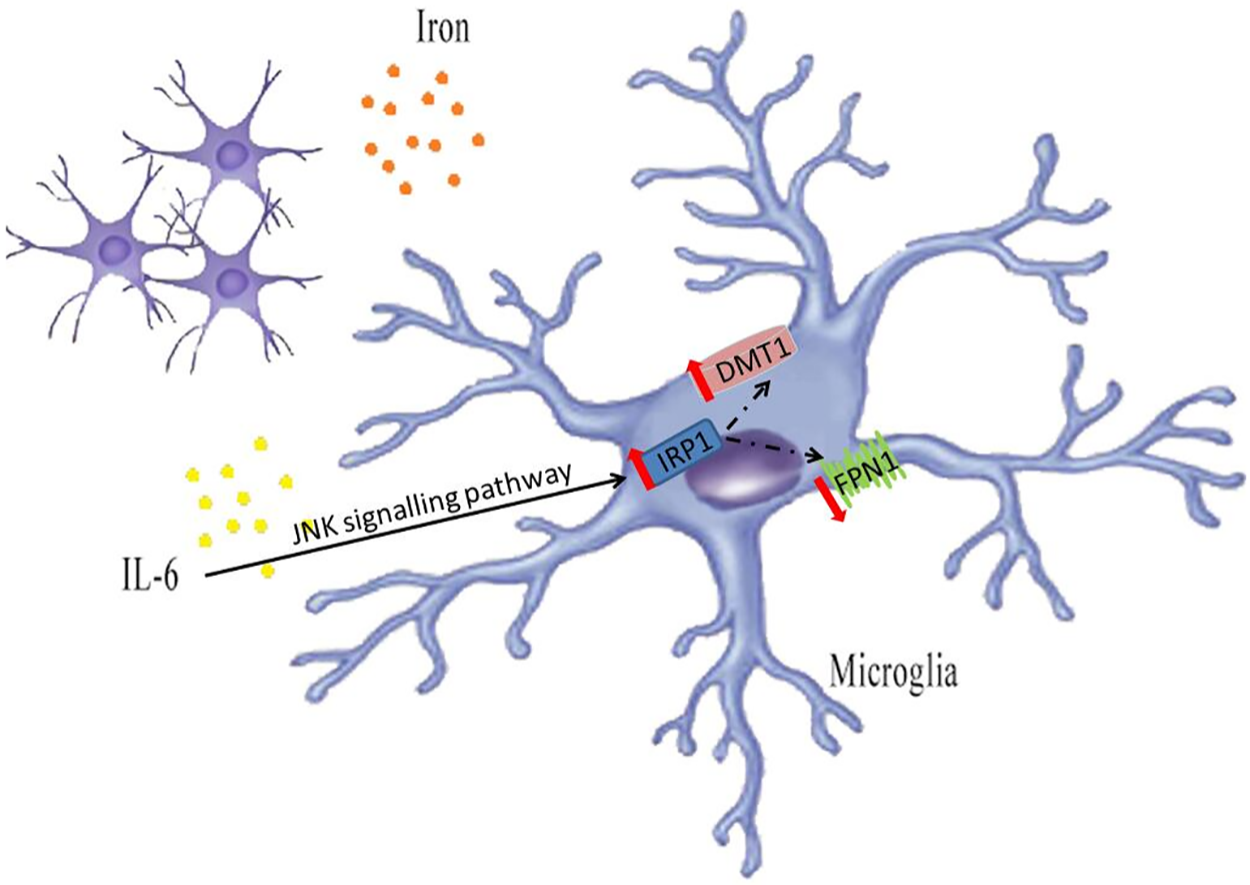
Graphical representation of the mechanism by which iron load can increase IL-6 mRNA expression, and IL-6 can regulate iron related proteins in BV2 cells. Iron load can increase IL-6 mRNA expression in BV2 cells. IL-6 up-regulates DMT1 expression and down-regulates FPN1 expression by IRP1 activation in BV2 microglial cells through JNK signaling pathways.

The present study showed that iron load could increase IL-6 mRNA expression in BV2 microglial cells. IL-6 might cause iron accumulation in BV2 microglial cells by up-regulating IRP1 and DMT1 expression and down-regulating FPN1 expression through JNK signaling pathways (Fig 5).

PD is the most common neurodegenerative movement disorder, affecting about 1% of people aged 65 or older worldwide [10]. Inflammation and abnormal iron deposition in the SN have proven roles in the pathogenesis of PD. However, the interplay between iron and inflammation in PD is complex. Neuronal death could induce inflammation by releasing apoptotic and necrotic cellular factors that are recognized by innate immune cells [11]. Extracellular α-synuclein might also induce inflammation through the activation of Toll-like receptors found on the surface of innate immune cells such as microglia [12]. Microglia are typically categorized into ‘classically’ activated macrophages (M1 type) or ‘alternatively’ activated macrophages (M2 type) [13]. In PD, activated macrophages release various pro-inflammatory cytokines such as TNF-α, IL-1β, and IL-6 and oxidative metabolites that can cause cytotoxicity [14]. TNF-α and IL-1β markedly increase in the nigrostriatal DA regions and cerebrospinal fluid of patients with PD [15]. In vivo and in vitro experiments have shown that TNF-α and IL-1β may induce dopaminergic neuron loss and contribute to PD pathogenesis [16]. Our group has proven that TNF-α and IL-1β released by microglia, especially under conditions of iron load, might contribute to iron accumulation in ventral mesencephalon neurons [7]. Microglia are essential sources of IL-6 in the CNS, and IL-6 levels in the cerebrospinal fluid of patients with PD are elevated [17]. A recent meta-analysis demonstrated higher peripheral concentrations of IL-6 in patients with PD, strengthening clinical evidence that PD is accompanied by an inflammatory response [18]. During inflammation and infection, IL-6 directly regulates hepcidin expression [19]. Hepcidin plays an important role in regulating iron metabolism, and can downregulate FPN1 and alter other iron transport proteins such as transferrin receptor 1 [20].

Iron accumulation is a characteristic feature of degenerating regions in the PD brain [21]. Iron can mediate the generation of reactive oxygen species and related processes including glutathione consumption, protein aggregation, lipid peroxidation, and nucleic acid modification [22,23]. Excess reactive oxygen species cause serious oxidative stress and induce the aggregation of α-synuclein in PD [24,25]. IRP1 is considered a central regulator of cellular iron metabolism, mediating regulation of the synthesis of proteins required for the uptake, storage, and export of iron by binding to mRNA containing an iron responsive element (IRE). For example, FPN1 mRNAs contain IRE in the 5′ untranslated region (UTR), where binding of the IRP is believed to interfere with the initiation of translation, thus leading to decreased FPN1 expression [5]. IRP1 binds to the IRE in the 3′-UTR of DMT1+IRE mRNA, and can increase DMT1 stability, thus leading to increased DMT1 expression [26]. Increase in IRP1 binding activity could be a result of an increased affinity for the consensus IRE, or due to increased protein levels [5]. We observed that, after IL-6 treatment, IRP1 was up-regulated, which induced increased DMT1 expression and decreased FPN1 expression, leading to iron accumulation in microglia.

JNKs transfer phosphate groups to serine or threonine residues flanked by a carboxy-terminal proline and are components of a classical mitogen-activated protein kinase signaling cascade [27]. JNK activity is increased in post-mortem brain tissue from patients with PD [28, 29]. In a previous study, we found that the neurotoxin MPP^+^ induced cytotoxicity in MES23.5 cells through JNK activation [30]. Iron causes selective and progressive dopaminergic neuron degeneration, and microglial NOX2 activation potentiates the neurotoxicity through JNK activation [31]. Many potential compounds and molecules that inhibit JNK signaling have been investigated as potential therapeutic agents for PD-related neuroinflammation [32,33]. Iron can contribute to JNK activation in rats [34]. Patients with myelodysplastic syndromes with iron overload exhibited increased JNK expression [35]. Inhibition of JNK activation could regulate some iron-metabolism-related proteins, such as Ferritin H chain [36]. IL-6 could induce JNK signaling. The JNK signaling cascade regulates the death of neuronal cells and may represent a suitable therapeutic target for rational drug design in PD [37]. Our results suggest that inhibition of JNK signaling could reverse abnormal iron homoeostasis in microglia.

Past reports suggest that uncontrolled activation of microglia may be directly toxic to neurons [38]. Our results provide new insights into the role of microglia in PD. Under iron overload, inflammatory cytokines, such as IL-6, released by microglia, likely activate IRP1, which up-regulates DMT1 expression and down-regulates FPN1 expression, leading to iron accumulation. This regulatory action of IL-6 on iron metabolism in microglia likely occurs via JNK signaling pathways.

## Supporting information

S1 Fig Effect of IL-6 and SP600125 on cell viability determined by MTT assays. A: MTT analysis of cell viability with IL-6 (30 ng/ml, 50 ng/ml and 100 ng/ml) treatment. B:MTT analysis of cell viability with SP600125 (10 and 20 μmol/L) treatment. Each bar represents the mean ± S.E.M of 4 independent experiments. We used the MTT assay to test the doses of IL-6 and SP600125. The viability of BV2 cells treated with different concentrations of IL-6 (30, 50, and 100 ng/ml) for 24 h was unchanged compared with the control (P > 0.05, n = 4), see Fig. S1A. The viability of BV2 cells treated with SP600125 (10 and 20 μmol/L) for 24 h was unchanged compared with the control (P > 0.05, n = 4), see Fig. S1B.We chose the minimum doses of 30 ng/ml IL-6 and 10 μmol/L SP600125 for subsequent experiments.
(TIF)
