# Supplementary figures and images for: Interleukin-6 regulates iron-related proteins through c-Jun N-terminal kinase activation in BV2 microglial cell lines
Source: PLoS One. 2017 Jul 3;12(7):e0180464. doi: 10.1371/journal.pone.0180464 (PMC5495437; doi:10.1371/journal.pone.0180464)

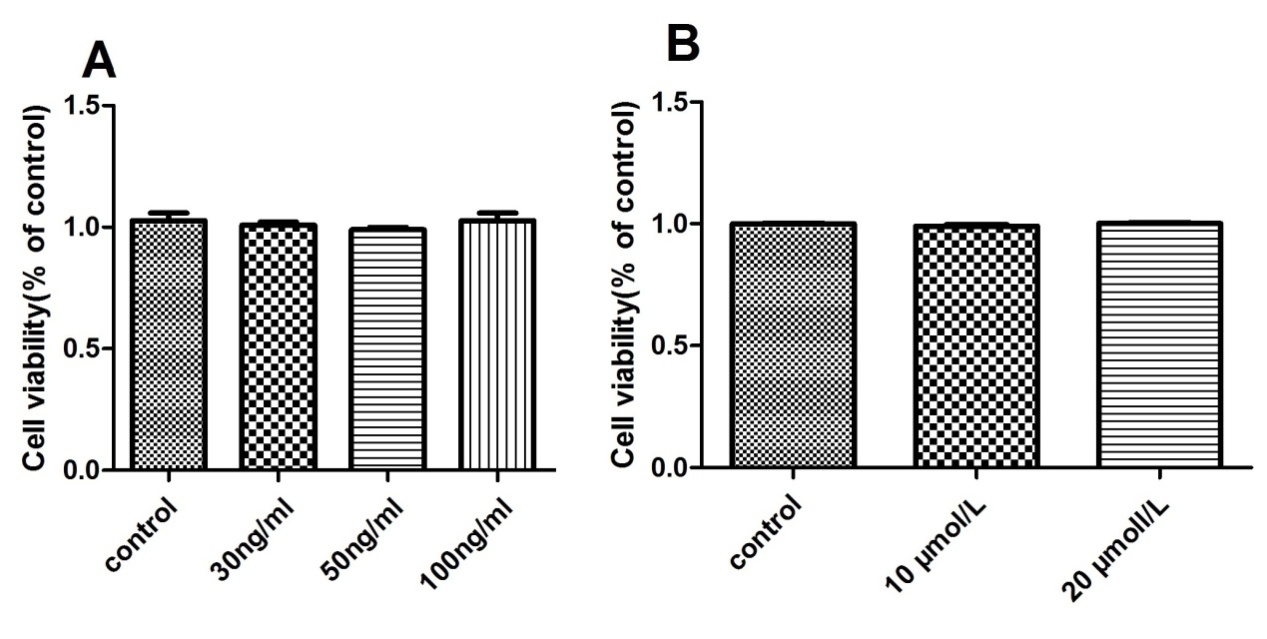

Supplement: S1 Fig — A: MTT analysis of cell viability with IL-6 (30 ng/ml, 50 ng/ml and 100 ng/ml) treatment. B:MTT analysis of cell viability with SP600125 (10 and 20 μmol/L) treatment. Each bar represents the mean ± S.E.M of 4 independent experiments. We used the MTT assay to test the doses of IL-6 and SP600125. The viability of BV2 cells treated with different concentrations of IL-6 (30, 50, and 100 ng/ml) for 24 h was unchanged compared with the control (P > 0.05, n = 4), see Fig. S1A. The viability of BV2 cells treated with SP600125 (10 and 20 μmol/L) for 24 h was unchanged compared with the control (P > 0.05, n = 4), see Fig. S1B.We chose the minimum doses of 30 ng/ml IL-6 and 10 μmol/L SP600125 for subsequent experiments. (TIF) [file pone.0180464.s001.tif]
